# Northwest Texas Medical Association

**Published:** 1894-10

**Authors:** 


### Society Notes.

### NORTHWEST TEXAS MEDICAL ASSOCIATION.

BOWIE, TEXAS, September 20, 1894.

DEAR DOCTOR:—The next semi-annual meeting of the Northwest Texas Medical Association convenes in the city of Bowie, on the 9th day of October, and we take this method of extending to you an earnest invitation to meet with us, and give us the benefit of your counsels as well as the encouragement of your presence.

Our object is to exchange and interchange thoughts on all medical subjects, as well as to engender a more extensive acquaintance and fraternal feeling among the physicians of Northwest Texas.

Our Association is young, but thoroughly in earnest. There will be several very able papers read at the coming meeting that we know of, and hope for some volunteer papers, and would be glad to have you prepare and read us a paper, or report an interesting case.

[Program omitted.—ED.]

Hoping to see you at Bowie on the 9th of October, I am, yours fraternally, W. L. YORK, M. D., President,

WM. C. DUNAWAY, Secretary.

SPECIAL RATES.

A special rate of one and one-third fare for round trip tickets has been secured, and reduced rates have been tendered the Association by the hotels of the city.

It is expected that this will be the grandest convocation of physicians ever held in Northwest Texas, and we most earnestly solicit your presence.

Bowie's most generous huspitality, in all its fullness, will be extended the Association. H. RILEY,

Chairman Committee of Arrangment.

